# Effect of Previous INR Control during VKA Therapy on Subsequent DOAC Adherence and Persistence, in Patients Switched from VKA to DOAC

**DOI:** 10.1055/a-2168-9378

**Published:** 2023-10-09

**Authors:** Tessa Elling, Eelko Hak, Jens H. Bos, Vladimir Y. I. G. Tichelaar, Nic J. G. M. Veeger, Karina Meijer

**Affiliations:** 1 Department of Hematology, University Medical Centre Groningen, University of Groningen, Groningen, The Netherlands; 2 Groningen Research Institute of Pharmacy, Unit of Pharmacotherapy, Epidemiology and Economy, University of Groningen, Groningen, The Netherlands; 3 Certe Thrombosis Service, Groningen, The Netherlands; 4 Department of Epidemiology, University of Groningen, University Medical Centre, Groningen, The Netherlands

**Keywords:** atrial fibrillation, venous thromboembolism, direct oral anticoagulant, persistence, adherence

## Abstract

**Introduction** Current guideline suggests a switch from vitamin K antagonist (VKA) to direct oral anticoagulant (DOAC) in patients with low time in therapeutic range (TTR < 70%). Poor international normalized ratio (INR) control may be the result of poor compliance, and might therefore be associated with subsequent DOAC intake. Therefore, this study evaluates the effect of previous TTR and other measures of INR control on DOAC nonadherence and nonpersistence, in patients who switched from VKA to DOAC.

**Methods** A total of 437 patients who switched from VKA to DOAC between 2012 and 2019 were included using data from Certe Thrombosis Service, IADB.nl pharmacy community database University Groningen, and Statistics Netherlands. DOAC prescriptions were used to determine nonadherence and nonpersistence. INR control (i.e., TTR, time under therapeutic range [TUR], and INR variability) was assessed during the last 180 days of VKA use. Multivariable regression models were applied to determine the association between INR control and DOAC nonpersistence/nonadherence.

**Results** On VKA, 67.7% of the patients had a TTR below 70%. DOAC nonpersistence was 39.8% (95% confidence interval [CI]: 33.4–45.5%) during a median follow-up of 34.4 months (interquartile range: 19.1–49.2). Approximately 80% of persistent patients were DOAC-adherent. Low TTR was not associated with DOAC nonpersistence (hazard ratio: 1.14, 95% CI: 0.69–1.87) and DOAC nonadherence (odds ratio: 1.38, 95% CI: 0.67–2.84), nor were TUR and INR variability.

**Conclusion** Previous INR control during VKA therapy is not associated with subsequent DOAC nonadherence and nonpersistence. This study suggests that INR control on VKA cannot, and therefore should not, be used for predicting DOAC adherence or persistence.

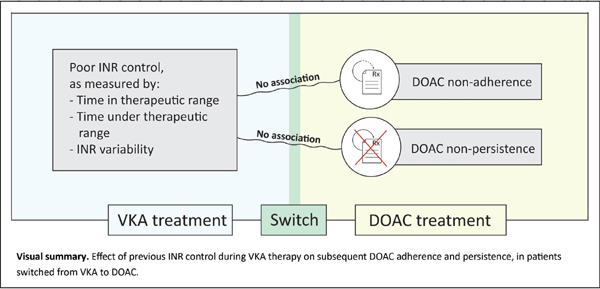

## Introduction

Oral anticoagulation therapy, including vitamin K antagonists (VKAs) and direct oral anticoagulants (DOACs), is widely used for the prevention and treatment of thromboembolism for many indications (e.g., venous thromboembolism [VTE], atrial fibrillation [AF]). 1 2 For many years, VKAs were the most frequently used anticoagulant. In these patients, frequent international normalized ratio (INR) monitoring and potential dose adjustments are needed, because of the narrow therapeutic window and variable dose–response relation of VKAs. To optimize anticoagulation therapy, a current guideline suggests that VKA-treated patients with AF and low INR time in therapeutic range (TTR < 70%) should switch to a DOAC because of their pharmacokinetic advantages and comparable efficacy. 1

DOACs are replacing VKAs as the main oral anticoagulant since their introduction in 2011. However, there are some concerns about the adherence and persistence on DOACs. Previous studies reported suboptimal adherence with an estimated adherence rate of 65 to 95%, as measured by usage of drug prescription databases. 3 4 5 6 7 In addition, approximately 30% of the patients were nonpersistent after DOAC initiation within a follow-up period of 2 to 4 years. 3 The pharmacokinetic advantages of DOACs, including the lack of INR monitoring due to their predictable dose–response curve, might contribute to these suboptimal adherence and persistence rates. In the Netherlands, thrombosis services manage VKA therapy by facilitating regular INR monitoring and potential dose adjustments. These regular contact moments are discontinued when patients switch from VKA to DOAC therapy.

Poorly taken anticoagulation is a major public health problem as it is associated with increased thromboembolic events, all-cause mortality, and higher health care costs. 8 Several risk factors have been identified for nonadherence and nonpersistence in patients treated with a DOAC, including a younger age, male sex, and a longer duration of anticoagulation therapy. 9 10 However, whether previous INR control is associated with DOAC intake in patients who have switched from VKA to DOAC is unclear. This information could impact the decision of physicians whether to switch patients from VKA to DOAC. Therefore, the aim of this study is to evaluate the effect of previous INR control on DOAC nonadherence and nonpersistence, in patients who had switched from VKA to DOAC therapy.

## Methods

### Data Sources and Study Population

This retrospective inception cohort study included adult patients who switched from VKA to DOAC for any anticoagulation indication. Relevant data were extracted from three databases, i.e., data from Certe Thrombosis Service, IADB.nl pharmacy community database University Groningen (IADB.nl), and Statistics Netherlands (Dutch Central Bureau of Statistics, CBS). The extracted data covers a study period between January 1, 2012 and December 31, 2019.

The CBS is a government agency that gathers data from a variety of sources and combines them at an individual level. The main purpose of CBS is to produce relevant national statistics for policy-makers and scientific researchers. For this study, we used data on mortality and emigration from the CBS.

Certe Thrombosis Service manages all VKA therapy for noninstitutionalized patients and patients of nursing homes in a large area in the north of the Netherlands. From the Certe database, we collected data on VKA treatment (indication, target range, and start and end date of VKA therapy) and the INR measurements (INR values, date of INR measurement, and method of INR measurement). INR values were measured at home, at the outpatient clinic or by the patients themselves, depending on a patient's mobility and cognitive functions. In line with the Thrombosis Services treatment protocol, patients with INR values measured by a health care professional at home were defined as frail, in contrast to the patients with INR measurements at the outpatient clinic and the patients who measured the INR values by themselves.

IADB.nl is a prescription database consisting of approximately 120 community pharmacies in the north of the Netherlands, covering an estimated population of 1.2 million patients. 11 Under the *Pharmlines initiative* , several databases have been linked via CBS as a trusted third party (TTP; see further). 7 Data extracted from the IADB.nl database included patient characteristics (i.e., patient sex and age in years at the time of switch to DOAC [index date] and prescription data of DOAC therapy and comedication [i.e., type, daily frequency, and date and number of tablets dispensed]). Patients who were taking antiplatelet therapy (APT) at the time of index date were classified as concurrent APT users. The index date was defined as the date of the first DOAC prescription after VKA therapy. The Anatomical Therapeutic Chemical codes (ATC codes) of the World Health Organization were used for medication identifications. The ATC codes extracted for this study are presented in the Supplementary Material ( Supplementary Table S1 , available in the online version).

The CBS acted as a TTP and was responsible for combining all relevant data from the three databases. After removing all identifying information, authorized/supervised access to the final database was granted to T.E. (first author). This procedure is in accordance with the Dutch General Data Protection Regulation. In this, the privacy of all study subjects was ensured.

### INR Control

This study focused on the association between previous INR control and subsequent DOAC nonadherence and nonpersistence, in patients who had first switched from VKA to DOAC therapy. INR control was assessed by TTR, TUR (i.e., time under the therapeutic range), and INR variability in the last 180 days of VKA use. TTR and TUR were calculated using the Rosendaal interpolation method. 12 Subsequently, TTR was dichotomized in low and adequate TTR with a cut-off value of 70%, as defined by a current guideline. 1 The method of Fihn et al was used to calculate INR variability. 13 A lower value represents lower INR variability, which is associated with a favorable risk profile for bleeding and thrombosis. 14 15 16 For reliable calculations, only patients with three or more INR values within the abovementioned 180 days and a time interval between INR values less than 56 days were included in this study. 17

### DOAC Persistence

DOAC persistence and adherence were calculated after index date, using data from the IADB.nl database (i.e., type, daily frequency, and date and number of DOAC tablets dispensed). Patients were nonpersistent on DOAC therapy if they switched to another anticoagulant (i.e., low-molecular-weight heparin [LMWH] or VKA) or when they discontinued their DOAC therapy (i.e., a gap of more than 180 days after the date of the last DOAC prescription) after index date. 18 The date of DOAC discontinuation was set at the date of the first prescription of VKA or LMWH (in patients switched from DOAC to another anticoagulant after index date) or at the end of the last DOAC prescription (in patients discontinuing anticoagulation therapy after index date).

Information about mortality, emigration, and usage of comedication after discontinuation of DOAC therapy was used to censor patients and to avoid misclassification of DOAC nonpersistence. Follow-up ended prematurely (i.e., before December 31, 2019) when patients died or emigrated.

### DOAC Adherence

DOAC adherence was evaluated by measuring the proportion of days covered (PDC), which is defined as the number of doses dispensed in relation to the dispensing period. To calculate PDC, at least two DOAC prescription fills are needed in a given time interval. In this study, the PDC was calculated in persistent patients during a 180-day period after index date. 18 We used an adapted version of the R Code published by Bijlsma et al to calculate PDC ( Supplementary Table S2 , available in the online version). 19

When calculating PDC, overlap between prescription dates was allowed. If a DOAC prescription was refilled before the end of the previous prescription, the new prescription was assumed to begin after the end of the previous prescription. This stockpiling in individual patients was allowed during 1 year. From then on, the excess of pills carrying on to the next year were not taken into account. Patients with a PDC of at least 90% were defined as high-adherent. Intermediate and low adherence were defined as a PDC between 66 and 90% and a PDC below 66%, respectively. 20 Another commonly used definition for DOAC adherence (i.e., low [PDC ≤ 50%], intermediate [PDC between 50 and 80%], and high [PDC ≥ 80%] adherence) was not used in this study, because of the very limited number of patients with a PDC value below 80% (see the “Results” section).

We considered DOACs interchangeable in the adherence and persistence calculations. If patients switched to another DOAC, the calculation of DOAC adherence and persistence included the prescriptions of both drugs.

### Statistical Analyses

Statistical analyses were performed using R version 4.1.2 (R Foundation for Statistical Computing, Vienna, Austria). Descriptive statistics were generated for all variables. Comparison of continuous variables was performed with the Student's independent samples T-test or Mann–Whitney U test, as appropriate. In addition, categorical data were compared using the Chi-square test or the Fisher's exact test, as appropriate.

Kaplan–Meier curves were used to evaluate nonpersistence for all patients and stratified by TTR, TUR, and INR variability. Patients at the end of follow-up (i.e., December 31, 2019) and patients who were lost to follow-up due to mortality or emigration were censored. Median follow-up was calculated with the inverse Kaplan–Meier approach. The association between INR control (i.e., TTR, TUR, and INR variability) and DOAC nonpersistence was evaluated using Cox proportional hazard models.

Differences due to the underlying type of DOAC nonpersistence, i.e., “switch to an anticoagulant other than DOAC” and “discontinuation of any anticoagulation therapy” were evaluated using competing risk analyses based on the Fine and Gray method.

Multinomial logistic regression analyses were performed to evaluate the association between INR control and DOAC nonadherence after index date during the following time intervals: 0–180 days, 0–1 year, 0–2 years, and 0–3 years. DOAC adherence was classified as low, intermediate, and high adherence, as previously described.

The effect of possible confounders (i.e., age, sex, VKA type, duration of VKA therapy, frailty, DOAC type, DOAC switch, and APT use) was evaluated in the multivariable Cox and logistic regression analyses with a backward elimination strategy. In this, variables with a *p* -value of 0.20 in univariate analyses were included. Furthermore, first-order interaction was considered (age, sex, duration of VKA therapy, time period). The variable time period was created by stratifying the patients into tertiles based on the index date.

To examine the robustness of our findings, we performed three additional sensitivity analyses. First, as INR values might be unstable at the beginning of VKA therapy, we stratified patients by duration of VKA treatment (i.e., patients treated with VKA for less than 6 months, compared to patients treated with VKA for more than 6 months). The INR values during the first 3 months after VKA initiation were excluded in the patients treated with VKA for more than 6 months. Secondly, a more strict definition of DOAC nonpersistence (i.e., a gap of more than 90 days at the end of the last DOAC prescription) was used to evaluate the association between INR control and DOAC nonpersistence. Third, a post-hoc sensitivity analysis was performed limited to patients with AF. Furthermore, a post-hoc subgroup analysis was performed, in which patients were stratified by DOAC frequency.

## Results

### Patient Characteristics

**Fig. 1:**
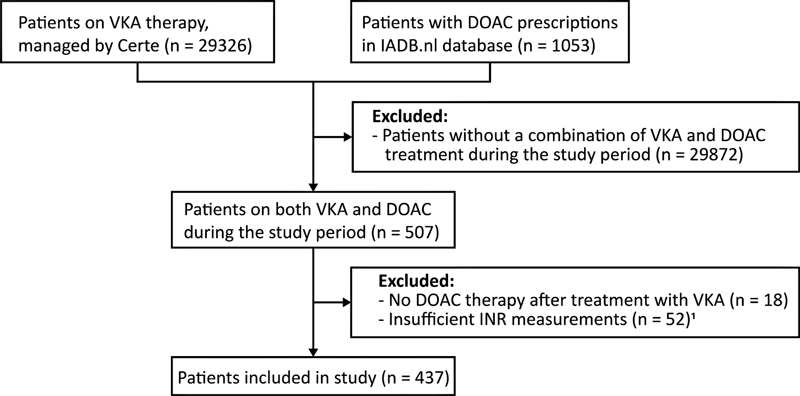
Flowchart. DOAC, direct oral anticoagulant; INR, international normalized ratio; VKA, vitamin K anticoagulant. ^1^ Patients with less than three INR values during the study period or patients with a time interval between INR values larger than 56 days.

We included a total of 437 patients with a median follow-up of 34.4 months (interquartile range [IQR]: 19.1–49.2] after their first DOAC prescription. ( Fig. 1 ). The mean age of this population was 69.6 ± 12.6 years; 238 (54.5%) patients were male. AF was the most common indication for anticoagulation therapy ( *n* = 356, 81.8%). Most patients previously used acenocoumarol ( *n* = 410, 93.8%) and had a low TTR (median: 57.8, IQR: 41.1–73.7) and a median TUR of 11.9 (IQR: 1.1–25.0). INR variability ranged from 0.03 to 4.48, with a median of 0.21 (IQR: 0.14–3.08). After index date, patients switched to apixaban ( *n* = 142, 32.5%), dabigatran ( *n* = 126, 28.8%), or rivaroxaban ( *n* = 149, 34.1%), and less frequently to edoxaban ( *n* = 20, 4.6%) ( Table 1 ). From 2012 to 2015, dabigatran was the most commonly prescribed DOAC ( *n* = 46, 61.3%). Rivaroxaban ( *n* = 136, 37.6%) and apixaban ( *n* = 126, 34.8%) were more frequently prescribed from 2015 to 2019.

**Table 1:** Patient characteristics

Patient characteristic	All patients ( *n* = 437)
Age (y), mean ± SD	69.6 ± 12.6
Male, no. (%)	238 (54.5)
Indication anticoagulation therapy a , no. (%)	
Atrial fibrillation	356 (81.8)
Venous thromboembolism	57 (13.1)
Other	22 (5.0)
VKA treatment b , no. (%)	
Acenocoumarol	410 (93.8)
Phenprocoumon	27 (6.2)
VKA target range b , no. (%)	
2.0–3.0	420 (96.1)
3.0–3.5	17 (3.9)
Duration of VKA therapy (mo), median [IQR]	30.7 [6.9–60.5]
Patients with previous periods of VKA treatment, no. (%)	34 (7.8)
Frailty c , no. (%)	
Frail patients	111 (25.4)
Nonfrail patients	326 (74.6)
DOAC treatment, no. (%)	
Apixaban	142 (32.5)
Dabigatran	126 (28.8)
Edoxaban	20 (4.6)
Rivaroxaban	149 (34.1)
Concurrent APT use at index date, no. (%)	43 (9.8)
TTR, median [IQR]	57.8 [41.1–73.7]
≥70%, no. (%)	141 (32.3)
<70%, no. (%)	296 (67.7)
INR variability, median [IQR]	0.25 [0.16–0.42]
TUR, median [IQR]	11.9 [1.1–25.0]

Abbreviations: APT, antiplatelet therapy; DOAC, direct oral anticoagulant; INR, international normalized ratio; TTR, time in therapeutic range; TUR, time under therapeutic range; VKA, vitamin K antagonist; VTE, venous thromboembolism.

a Data available from 435 patients.

b Assessed during the last 180 days of VKA use.

c Patients with INR values measured by a health care professional at home were defined as frail, in contrast to patients with INR measurements at the outpatient clinic ( *n* = 248) or INR measurements by the patient themselves (i.e., self-testing, *n* = 78).

### DOAC Nonpersistence

**Fig. 2:**
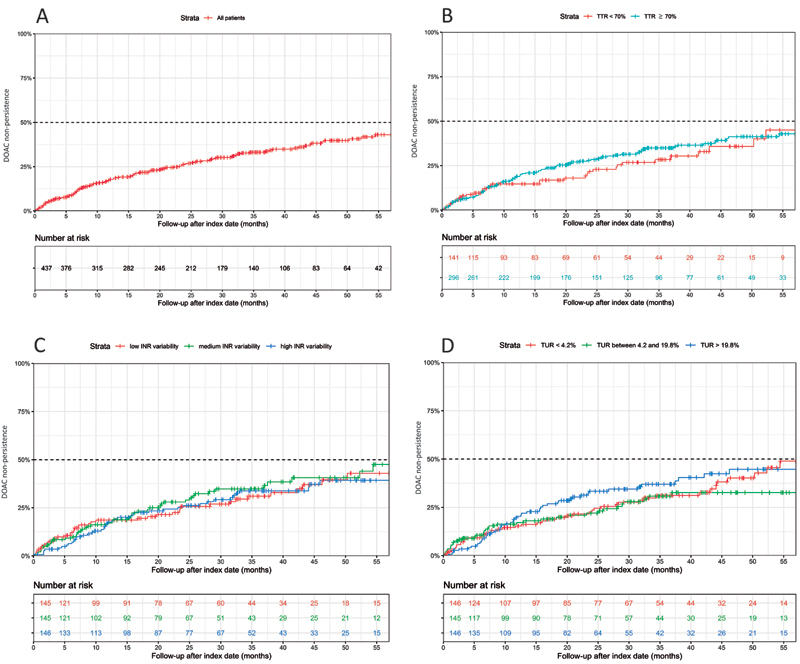
DOAC nonpersistence. DOAC nonpersistence evaluated in all patients ( **A** ) and stratified by TTR ( **B** ), INR variability ( **C** ), and TUR ( **D** ). DOAC, direct oral anticoagulant; INR, international normalized ratio; TTR, time in therapeutic range; TUR, time under therapeutic range.

In total, 17.8% (95% confidence interval [CI]: 14.0–21.4%) of the patients had discontinued their DOAC therapy after 1 year of follow-up. These nonpersistence rates increased over time with percentages of 26.6% (95% CI: 21.9–31.0%), 33.2% (95% CI: 27.9–38.1%), and 39.8% (95% CI: 33.4–45.5%) after 2, 3, and 4 years of follow-up, respectively ( Fig. 2A ). More specifically, after 1 year of follow-up, 7.0% (95% CI: 4.2–9.6%) had discontinued their anticoagulation therapy and 11.6% (95% CI: 8.5–14.7%) of the patients switched from DOAC to LMWH or back to VKA. After 4 years, more patients had discontinued their anticoagulation therapy (26.6%, 95% CI: 20.2–32.5%) compared to the patients who had switched to LMWH or back to VKA (17.9%, 95% CI: 13.3–22.3%). During the study period, 37 patients (8.5%) switched to another DOAC.

The nonpersistence rates differed between the once daily and twice daily dosed DOACs. After 1 year of follow-up, 11.2% (95% CI: 5.8–16.2%) of the patients with once daily dosed DOACs had discontinued their DOAC therapy, compared to 21.3% (95% CI: 16.0–26.3%) of the patients with twice daily dosed DOACs. After 4 years of follow-up, these percentages were 36.3 % (95% CI: 23.6–49.0%) and 42.2% (95% CI: 34.4–49.1%), respectively.

As shown in Fig. 2 , Table 2 , and Supplementary Table S3 (available in the online version), previous INR control on VKA, expressed as TTR, TUR, and INR variability, was not associated with subsequent DOAC nonpersistence. In this, differences in DOAC nonpersistence between patients with different levels of INR control could not be established. Potential effect modification by age, sex, duration of VKA therapy, and time period was addressed in the Cox regression analyses. No significant effect modification was observed. These findings were confirmed in the sensitivity analyses ( Supplementary Tables S4–S6 , available in the online version). In addition, there was no relevant association between INR control and the nonpersistence subtypes (“prematurely stopping any anticoagulation therapy” and “switch from DOAC to VKA or LMWH” after index date), as evaluated by competing risk analyses ( Table 3 ).

**Table 2:** Association between INR control and DOAC nonpersistence

Variable	Crude HR (95% CI)	Adjusted HR (95% CI) a
TTR		
≥70%	Reference	Reference
<70%	1.07 (0.74–1.54)	1.10 (0.73–1.64)
TUR		
<4.2%	Reference	–
≥4.2% and <19.8%	0.91 (0.60–1.39)	
≥19.8%	1.15 (0.78–1.70)	
INR variability		
Low INR variability	Reference	Reference
Medium INR variability	1.13 (0.76–1.70)	1.06 (0.70–1.62)
High INR variability	0.95 (0.63–1.43)	0.94 (0.60–1.47)

Abbreviations: CI, confidence interval; HR, hazard ratio; INR, international normalized ratio; TTR, time in therapeutic range; TUR, time under therapeutic range.

Note: Cox regression analyses evaluating the association between INR control and DOAC nonpersistence, including 437 patients.

a Adjusted for VKA type and DOAC type. This best-fitted multivariable model (based on an elimination strategy) did not include time under therapeutic range, to avoid of collinearity. The analyses evaluating possible confounders are presented in the Supplementary Table S3 (available in the online version).

**Table 3:** Association between INR control and DOAC nonpersistence, with subdivision into subtypes

	Discontinuation of any anticoagulation therapy ( *n* = 77)	Switch to another anticoagulant than DOAC ( *n* = 64)
Variable	SHR (95% CI)	SHR (95% CI)
TTR		
≥70%	Reference	Reference
<70%	1.14 (0.69–1.87)	1.02 (0.60–1.74)
TUR		
<4.2%	Reference	Reference
≥4.2% and <19.8%	0.72 (0.39–1.33)	1.08 (0.60–1.92)
≥19.8%	1.56 (0.95–2.58)	0.74 (0.40–1.36)
INR variability		
Low INR variability	Reference	Reference
Medium INR variability	0.94 (0.54–1.64)	0.83 (0.46–1.50)
High INR variability	1.30 (0.76–2.21)	0.82 (0.45–1.47)

Abbreviations: CI, confidence interval; INR, international normalized ratio; SHR, subdistribution hazard ratio; TTR, time in therapeutic range; TUR, time under therapeutic range.

Note: Competing risk model using the Fine and Gray method to evaluate the association between INR control and DOAC nonpersistence subtypes. Crude estimates are presented.

Compared to persistent patients, nonpersistent patients were older (mean: 70.5 years ± 11.7 vs. 67.7 years ± 14.3), treated with a VKA for a shorter period of time (17.6 months, IQR: 4.5–51.4 vs. 36.0 months, IQR: 9.8–63.2), and were more frequently treated with phenprocoumon before index date (11.3 vs. 3.7%) and with dabigatran after index date (41.1 vs. 23.0%) ( Table 4 ).

**Table 4:** Differences between persistent and nonpersistent patients

Patient characteristic	Persistent patients ( *n* = 296)	Nonpersistent patients ( *n* = 141)	*p* -Value
Age (y), mean ± SD	70.5 ± 11.7	67.7 ± 14.3	0.041
Male, no. (%)	165 (55.7)	73 (51.8)	0.499
Primary indication anticoagulation therapy a , no. (%)			
AF	236 (80.0)	120 (85.7)	0.664
VTE	41 (13.9)	16 (11.4)	
Other	18 (6.1)	4 (1.4)	
VKA treatment b , no. (%)			
Acenocoumarol	285 (96.3)	125 (88.7)	0.004
Phenprocoumon	11 (3.7)	16 (11.3)	
VKA target range b , no. (%)			
2.0–3.0	285 (96.3)	135 (95.7)	0.994
2.5–3.5	11 (3.7)	6 (4.3)	
Duration of VKA therapy (mo), median [IQR]	36.0 [9.8–63.2]	17.6 [4.5–51.4]	0.008
Frailty c , no. (%)			
Frail patients	78 (26.4)	33 (23.4)	0.586
Nonfrail patients	218 (73.6)	108 (76.6)	
DOAC treatment at index date d , no. (%)			
Apixaban	98 (33.1)	44 (31.2)	<0.001
Dabigatran	68 (23.0)	58 (41.1)	
Rivaroxaban	110 (37.2)	39 (27.7)	
DOAC switch after index date, no. (%)			
Switch	23 (7.8)	15 (10.6)	0.661
No switch	273 (92.2)	126 (89.4)	
Concurrent APT use at index date, no. (%)	31 (10.5)	12 (8.5)	0.637
TTR b			
<70%, no. (%)	194 (65.6)	102 (72.3)	0.190
≥70%, no (%)	102 (34.5)	39 (27.7)	
TUR b , median [IQR]	11.7 [1.1–22.8]	13.9 [2.2–26.7]	0.392
INR variability b , median [IQR]	0.26 [0.16–0.42]	0.24 [0.18–0.43]	0.660

Abbreviations: APT, antiplatelet therapy; DOAC, direct oral anticoagulant; INR, international normalized ratio; TTR, time in therapeutic; TUR, time under the therapeutic range; VKA, vitamin K therapy; VTE, venous thromboembolism.

a Data available from 435 patients for the two groups combined.

b Assessed during the last 180 days of VKA use.

c Patients with INR values measured by a health care professional at home were defined as frail, in contrast to patients with INR measurements at the outpatient clinic or INR measurements by the patient themselves (i.e., self-testing).

d Data about DOAC treatment “edoxaban” are not shown because of the limited number of patients in this category.

In Cox regression analyses, treatment with dabigatran was associated with a higher risk of DOAC nonpersistence, compared to patients treated with apixaban (hazard ratio [HR]: 1.60, 95% CI: 1.07–2.39). Furthermore, treatment with phenprocoumon before index date was associated with DOAC nonpersistence, compared to acenocoumarol (HR: 3.01, 95% CI: 1.77–5.10).

### DOAC Nonadherence

**Fig. 3:**
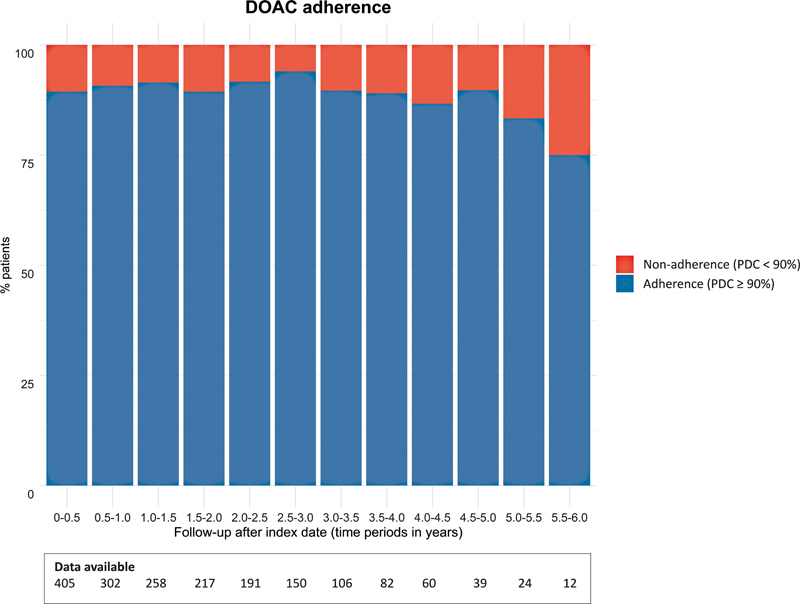
DOAC adherence. DOAC adherence evaluated during different time intervals after index date. Adherence was dichotomized into nonadherent (PDC < 90%) and adherent (PDC ≥ 90%). DOAC, direct oral anticoagulant; PDC, proportion of days covered.

During the first 180 days after index date, the proportion of patients with high adherence (i.e., PDC ≥ 90%), intermediate adherence (PDC between 66 and 90%), and low adherence (PDC ≤ 66%) were 363 (89.5%), 36 (8.9%), and 7 (1.7%), respectively. Because of the very limited number of patients with low and intermediate adherence during follow-up, a further dichotomization (i.e., adherent [PDC ≥ 90%] vs. nonadherent [PDC < 90%]) was used in the evaluation of nonadherence. During the entire follow-up, more than 80% of the patients remained adherent ( Fig. 3 ). The adherence rates remained similar after stratification by DOAC frequency. During the first 180 days after index date, the proportion of adherent patients was 130 (91.5%) for those with once-daily dosed DOACs and 214 (88.1%) for patients with twice daily dosed DOACs, respectively.

TTR, TUR, and INR variabilities were not associated with DOAC nonadherence during the time intervals 0–1 year, 0-–2 years, and 0–3 years after index date ( Table 5 ). In this, higher odds ratios (ORs) were seen when time periods increased. However, these ORs were accompanied by wide CIs not reaching statistical significance. Potential effect modifications by age, sex, duration of VKA therapy, and time period were addressed in the logistic regression analyses. No significant effect modification was observed. Sensitivity analyses confirmed the lack of association between INR control and DOAC nonadherence ( Supplementary Tables S7 and S8 , available in the online version). Furthermore, a more strict definition of DOAC nonpersistence (i.e., a gap of more than 90 days at the end of the last DOAC prescription) did not affect the proportion of patients being adherent.

**Table 5:** INR control and DOAC nonadherence

	Crude OR				Adjusted OR a			
Variable	0–6 months ( *n* = 406)	0–1 year ( *n* = 302)	0–2 years ( *n* = 217)	0–3 years ( *n* = 150)	0–6 months ( *n* = 406) b	0–1 year ( *n* = 302) c	0–2 years ( *n* = 217) b	0–3 years ( *n* = 150) d
TTR								
≥70%	Reference	Reference	Reference	NA	Reference	Reference	Reference	NA
<70%	1.38 (0.67–2.84)	1.29 (0.55–3.01)	1.86 (0.60–5.73)		1.72 (0.79–3.76)	1.84 (0.72–4.72)	2.05 (0.63–6.72)	
TUR								
<4.2%	Reference	Reference	Reference	Reference	NA	NA	NA	NA
≥4.2% and <19.8%	0.48 (0.21–1.12)	0.62 (0.23–1.65)	0.94 (0.30–2.94)	4.00 (0.40–39.82)				
≥19.8%	0.87 (0.43–1.79)	1.09 (0.47–2.56)	1.48 (0.52–4.20)	9.56 (1.13–80.73) e				
INR variability								
Low INR variability	Reference	Reference	Reference	Reference	Reference	Reference	Reference	Reference
Medium INR variability	0.61 (0.28–1.35)	0.92 (0.38–2.19)	1.00 (0.33–3.02)	1.21 (0.23–6.31)	0.54 (0.24–1.21)	0.88 (0.35–2.24)	0.86 (0.28–2.66)	0.84 (0.14–4.92)
High INR variability	0.73 (0.35–1.53)	0.59 (0.23–1.50)	0.97 (0.33–2.82)	1.51 (0.34–6.65)	0.58 (0.26–1.29)	0.47 (0.17–1.33)	0.76 (0.24–2.34)	0.83 (0.17–4.04)

Abbreviations: CI, confidence interval; INR, international normalized ratio; NA, not applicable; PDC, proportion of days covered; TTR, time in therapeutic range.

Note: Logistic regression analyses evaluating the association between INR control and DOAC nonadherence. Nonadherence was defined as a PDC value below 90%. Because of collinearity, TUR was not included in the adjusted model. The analyses evaluating other possible confounders are presented in the Supplementary Table S8 (available in the online version).

a To avoid collinearity, TUR was not included in the adjusted models. Different models were used to get a best-fitted model (based on a backward elimination strategy), including:

b Inclusion of TTR and INR variability only.

c Inclusion of TTR and INR variability, adjusted for age and DOAC type.

d Inclusion of TTR and INR variability, adjusted for sex.

e *p* -Value < 0.05.

There were no differences observed between adherent (PDC ≥ 90%) and nonadherent patients (PDC < 90%) ( Supplementary Table S9 , available in the online version). However, patients aged between 67 and 80 years seemed to be more adherent, compared to patients younger than 67 years (OR: 0.34, 95% CI: 0.14–0.82), during the 0–1 year time interval. In the same time interval, treatment with dabigatran was associated with DOAC nonadherence, compared to apixaban (OR: 2.84, 95% CI: 1.09–7.35). During the 0–3 years time interval, male sex was associated with DOAC nonadherence (OR: 11.84, 95% CI: 1.46–96.3) ( Table 5 and Supplementary Table S10 , available in the online version).

## Discussion

This retrospective cohort study evaluated 437 patients on long-term anticoagulation therapy, who had switched from VKA to DOAC. During VKA therapy, the majority of the patients were not well-controlled, as 67.7% of the patients had a TTR below 70%. After switch, 17.8% (95% CI: 14.0–21.4) of the patients had discontinued their DOAC therapy after 1 year of follow-up. Of these patients, 11.6% (95% CI: 8.5–14.7%) switched to an anticoagulant other than a DOAC. After 4 years of follow-up, 39.8% (95% CI: 33.4–45.5) of the patients no longer used a DOAC, with 17.9% (95% CI: 13.3–22.3%) of them switched to a VKA or a LMWH. In persistent patients, DOAC adherence was high during the entire follow-up. PDC values above 90% were observed in approximately 80% of the patients during the study period. In this study population, we found no association between previous INR control on VKA and subsequent nonpersistence and nonadherence on DOAC therapy.

### Comparison to Other Studies

#### DOAC Nonpersistence

Previous observational studies that also measured nonpersistence with the usage of a drug prescription database reported a large variation in nonpersistence. A meta-analysis, conducted by Ozaki et al, included 36 observational studies reporting DOAC persistence in patients with AF. They calculated a pooled proportion of 31% (95% CI: 28–35%) for DOAC nonpersistence for all follow-up durations (i.e., follow-up between 3 and 24 months). 3 More recent studies and studies evaluating patients with VTE reported nonpersistent rates varying from 5 to 60% during a follow-up period between 2 months and 4 years. 4 5 8 10 21 22 23 24 25 The persistence rate of the current study fits in this variation of DOAC nonpersistence. In the previous studies, lower persistence rates were seen in patients with a longer follow-up period, in anticoagulant-naïve patients and in patients treated with dabigatran. 3 10 21 In addition, different definitions of nonpersistence were used, varying between a gap of 30 to 180 days after the last prescription. Studies reporting a lower proportion of nonpersistence used more frequently a more conservative definition. 3 10

#### DOAC Nonadherence

Variation was also seen in observational studies evaluating adherence by calculating PDC. According to the meta-analysis of Ozaki et al, a 12-month overall pooled proportion of good adherence (i.e., PDC > 80%) was 68% in patients with AF. 3 More recent studies evaluating AF patients reported PDC values varying between 65 and 95%, with a follow-up duration varying between 3 months and 3 years. 4 5 6 7 PDC values above 90% were more frequently seen in patients with VTE. This suggests better adherence in VTE patients compared to patients with AF. 3 8 26 In addition, lower adherence rates were more frequently seen in patients with a longer observation time and in patients without previous VKA experience. 3

The current study reported higher adherence rates compared to the previous studies. For this, different reasons could be given. First, patient characteristics varied between the studies. The patients included in this study were experienced anticoagulation users, as they switched from VKA to DOAC. The majority of the patients in the other studies had no previous VKA experience. In these anticoagulant-naïve patients, lower adherence and persistence rates were observed, compared to patients who had switched from VKA to DOAC. 6 10

Second, the patient population selected for this study might be more frequently evaluated by a cardiologist or an internist at an outpatient clinic, as these physicians actively switch patients from VKA to DOAC. Frequent evaluations by a physician could influence patient behavior by improving DOAC adherence, as medication compliance and the importance of anticoagulation therapy might be discussed. High adherence rates were also seen in randomized clinical trials, possibly caused by intensive patient follow-up. 27 28 29 However, at these outpatient clinics, most patients are switched from VKA to DOAC without knowledge about previous INR control.

In this study, a very limited number of patients were defined as low (PDC values below 66%) or intermediate adherent (PDC values between 66 and 90%). 20 As a result, a further dichotomization (i.e., adherent [PDC ≥ 90%] and nonadherent [PDC < 90%]) was used in the evaluation of DOAC nonadherence. Contrarily, previous studies defined patients with PDC values below 80% as nonadherent. 4 5 6 7 However, considering the very limited number of patients in this subgroup (i.e., approximately 5% of the patients had a PDC value <80% during the different time intervals), we used a more strict definition of 90% or higher.

### Association Between INR Control and DOAC Intake

Two observational studies evaluated the association between TTR on VKA and subsequent DOAC intake, in patients who had switched from VKA to DOAC. First, Toorop et al found an association between low TTR and DOAC nonpersistence. 24 Second, Pundi et al reported an association between low TTR and DOAC nonadherence. 7 This is in contrast to our study, as we found no association between TTR and DOAC intake. This discrepancy could be attributed to the differences in study population. Both previous studies included only patients with AF as treatment indication. 7 24 Furthermore, the patients included in the study of Pundi et al used warfarin as VKA, were almost all male, and had lower TTR values (45.0 [IQR: 26.0–64.0] vs. 57.8 [41.1–73.7]). 7 The patients enrolled by Toorop et al were older (74.2 ± 9.5 vs. 69.6 ± 12.6), had a lower number of patients with a TTR value below 70% (56.0 vs. 67.7%), and used more frequently phenprocoumon (32.5 vs. 6.2%), compared to our study. 24 Our findings are in line with the study of Toorop et al, as we observed an association between previous phenprocoumon use and the risk of subsequent DOAC nonpersistence/nonadherence.

The methods to evaluate DOAC adherence and persistence were similar, as they used prescription databases with similar definitions. 7 24

### Association Between Various Factors and DOAC Intake

Similar to previous studies, we found an association between DOAC nonpersistence and the usage of dabigatran. 3 Patients on dabigatran experience more frequently adverse side-effects, which could be an explanation for the higher nonpersistence rates in these patients. 30 The use of phenprocoumon before the index date was also associated with DOAC nonpersistence, which might be a regional effect. In the north of the Netherlands, most patients use acenocoumarol as VKA treatment. In patients with reduced INR control, patients are regularly switched to longer acting phenprocoumon aiming to improve INR control. While our study findings indicate an association between phenprocoumon and subsequent DOAC nonpersistence, it is important to acknowledge that this association is probably caused by patient characteristics rather than attributed to phenprocoumon itself.

### Strengths and Limitations

This study has several strengths. Accepted measures for the calculation of DOAC adherence and persistence were used. 13 30 Also data about emigration, mortality, duration of anticoagulation therapy, and the use of co-medication after discontinuing DOAC therapy strengthen our definition of nonpersistence. Furthermore, the prescription records are nearly complete, because of the high patient-pharmacy commitment in the Netherlands.

There are also some limitations. Medication during hospitalization are not covered in the IADB.nl database, which might affect DOAC nonadherence. Nevertheless, we think this effect is small, as hospitalization might be the case for a limited number of patients. 31 DOAC nonpersistence is not affected by hospitalization, as we define nonpersistence as a gap of more than 180 days after the end of the last DOAC prescription. Most patients are not hospitalized for 180 days or longer. Second, unidentified confounders may have informed and helped the health care professional in the decision to switch a patient from a VKA to DOAC. Comorbidities, such as cognitive disorders or Parkinson's disease, bleeding history, and fall risk, are potential confounding factors. Furthermore, the adherence and persistence calculations based on prescription records do not evaluate the actual intake of the anticoagulants. Therefore, the adherence and persistence rates in this study might be an overestimation of the actual intake, as with all studies evaluating adherence and persistence using prescription records. Lastly, the power of this study might be affected by the limited number of patients in the nonadherent group.

Patients treated with VKA and subsequent DOAC therapy for a short period of time might have a temporary indication for anticoagulation therapy. However, the median duration of previous VKA and subsequent DOAC therapy of 68.4 months (IQR: 41.5–90.0) suggests a population with mostly a long-term indication for anticoagulation therapy in this study.

### Clinical Relevance

Although this study does not evaluate clinical outcomes, previous studies observed an association between poorly taken anticoagulation and increased thromboembolic events, all-cause mortality, and higher health care costs. 9 10 Although several variables affecting DOAC intake are described in previous studies, the association between INR control and DOAC intake remains unclear, in patients who had switched from VKA to DOAC. Based on our findings, there is no association between INR control and DOAC intake. Therefore, INR control on VKA cannot, and therefore should not, be used for predicting DOAC adherence or persistence. Patients with poor INR control on VKA can be suitable candidates for DOAC therapy. Other factors, either fixed or modifiable, should be taken into account in this evaluation (e.g., age, sex, duration, and type of anticoagulation therapy).

## Conclusion

Based on our findings, previous INR control on VKA is not associated with subsequent DOAC nonadherence and nonpersistence. Therefore, INR control on VKA cannot, and therefore should not, be used for predicting DOAC adherence or persistence. Accordingly, patients with poor INR control on VKA can be suitable candidates for DOAC therapy. We propose a more individual approach in the decision to switch a patient from VKA to DOAC, based on patient characteristics and medical history.

## Notes

**What is known about this topic?**

- Poorly taken anticoagulation is a major public health problem as it is associated with increased thromboembolic events, all-cause mortality, and higher health care costs.
- Current guideline suggests a switch from vitamin K antagonist (VKA) to direct oral anticoagulant (DOAC) in patients with low time in therapeutic range (TTR < 70%).
- It is unclear whether low INR control is associated with subsequent poor DOAC intake.

**What does this paper add?**

- INR control during VKA therapy is not associated with subsequent DOAC nonadherence nor nonpersistence.
- This study suggests that INR control on VKA cannot, and therefore should not, be used for predicting DOAC adherence or persistence.
