# Supplementary material for: Effect of Previous INR Control during VKA Therapy on Subsequent DOAC Adherence and Persistence, in Patients Switched from VKA to DOAC
Source: Thromb Haemost. 2023 Oct 9;124(8):778–90. doi: 10.1055/a-2168-9378 (PMC11259495; doi:10.1055/a-2168-9378)
Supplement: Supplementary file 1 — Supplementary Material [file 10-1055-a-2168-9378-s23040163.pdf]

Supplementary Table S1 ATC codes

ATC code	Medication[a]
A10	Hypoglycemic drugs
B01AA04	Phenprocoumon
B01AA07	Acenocoumarol
B01AC	Antiplatelet therapy
B01AE07	Dabigatran etexilate
B01AF01	Rivaroxaban
B01AF02	Apixaban
B01AF03	Edoxaban
C01BD01	Amiodarone
C03	Diuretics
C07	Beta-blocking agents
C08	Calcium channel blockers
C09	Agents acting on the renin–angiotensin system
C10	Lipid-modifying agents
JO1FA	Macrolides
J02AB	Imidazole derivatives
J02AC	Triazole derivatives
J05AE	HIV protease inhibitors
MO1AB	NSAIDs
M01AE	NSAIDs
N03AA02	Phenobarbital
N03AB02	Phenytoin
N03AF01	Carbamazepine
N05	Psycholeptics
N06A	Antidepressants
N06D	Antidementia drugs
R03	Drugs for obstructive airway diseases

Abbreviations: HIV, human immunodeficiency virus; NSAID, nonsteroidal anti-inflammatory drugs; NSRI, nonselective monoamine reuptake inhibitor; SSRI, selective serotonin reuptake inhibitors.
Note: The Anatomical Therapeutic Chemical (ATC) codes used to select medication form the IADB.nl pharmacy database.
[a]Medication type or group.

 © 2023. The Author(s).

Supplementary Table S2 R script for evaluating DOAC adherence

```
# dataset: pharm.dat
# meaning of the columns in pharm.dat:
   # ID represents the name of the patient
   # dispendate the date when the pills were dispensed
   # pillsn the number of pills a patient receives in that dispensing
   # dailydose the number of pills a patient is expected to take
# with less than 2 entries: here we remove them from the data
# Make a file that shows the number of entries for each ID
entries <- table(pharm.dat$ID)
entryIDs <- data.frame(as.numeric(names(entries)), as.vector(entries))
names(entryIDs) <- c("ID","entries")
entryIDs[entryIDs$entries > 1,]
# Make a new dataset with ID's with less than 2 entries deleted
pharm.dat2 <- pharm.dat[pharm.dat$ID %in% entryIDs$ID[entryIDs$entries > 1],]
rm(entryIDs)
# Calculate for each patient the dates in person-time since first entry and assign a number to each row for each patient
pharm.dat2$rownr <- pharm.dat2$persontime <- NA
for(i in unique(pharm.dat2$ID)) {
   mindate <- min(as.Date(pharm.dat2$dispendate[pharm.dat2$ID == i],format = "%Y-%m-%d"))
   pharm.dat2$persontime[pharm.dat2$ID == i] <- as.Date(pharm.dat2$dispendate[pharm.dat2$ID == i],format = "%Y-%m-
   %d") - mindate
   pharm.dat2$rownr[pharm.dat2$ID == i] <- 1:length(pharm.dat2$ID[pharm.dat2$ID == i])
}
# Sort dataset by ID and persontime
pharm.dat2 <- pharm.dat2[order(pharm.dat2$ID,pharm.dat2$persontime),]
## Calculate interval length, total days covered and adherence in each interval ##
pharm.dat2$adherence <- pharm.dat2$intlength <- pharm.dat2$totdays <- NA
pharm.dat2$remove <- 0
for(i in unique(pharm.dat2$ID)) {
   entries <- length(pharm.dat2$ID[pharm.dat2$ID == i])
   # calculate interval length
   pharm.dat2$intlength[pharm.dat2$ID == i & pharm.dat2$rownr < (entries-1)] <- (pharm.dat2$persontime[pharm.dat2
   $ID == i][3:entries]) - (pharm.dat2$persontime[pharm.dat2$ID == i][1:(entries-2)])
   # calculate total days covered by drug in each interval
   pharm.dat2$totdays[pharm.dat2$ID == i & pharm.dat2$rownr < (entries-1)] <- (pharm.dat2$pillsn[pharm.dat2$ID == i]
   [1:(entries-2)])/(pharm.dat2$dailydose[pharm.dat2$ID == i][1:(entries-2)]) +
   (pharm.dat2$pillsn[pharm.dat2$ID == i][2:(entries-1)])/(pharm.dat2$dailydose[pharm.dat2$ID == i][2:(entries-1)])
   # use a for-loop to deal with drug stockpiling
   extra <- 0 # set extra pills to 0 before the loop so pills don't go from one patient to an adjacent patient
   for(rows in 1:(length(pharm.dat2$ID[pharm.dat2$ID == i])-2)) {
   pharm.dat2$totdays[pharm.dat2$ID == i & pharm.dat2$rownr == rows] <- pharm.dat2$totdays[pharm.dat2$ID == i &
   pharm.dat2$rownr == rows] + extra
   ifelse(pharm.dat2$totdays[pharm.dat2$ID == i & pharm.dat2$rownr == rows]-pharm.dat2$intlength[pharm.dat2$ID == i
   & pharm.dat2$rownr == rows] > 0,
   extra <- pharm.dat2$totdays[pharm.dat2$ID == i & pharm.dat2$rownr == rows]-pharm.dat2$intlength[pharm.dat2$ID
   == i & pharm.dat2$rownr == rows],
   extra <- 0)
   # if more days are covered than the length of the interval, then assign the extra pill days to the 'extra' object
   # (which carries them over to the next row), and otherwise set extra to 0
   }
   # the last two rows for each patient get the remaining pill days plus any pill days carried forward from stockpiling
   pharm.dat2$totdays[pharm.dat2$ID == i & pharm.dat2$rownr == (entries-1)] <-
   sum(c(pharm.dat2$pillsn[pharm.dat2$ID == i & pharm.dat2$rownr > (entries-2)]/pharm.dat2$dailydose[pharm.dat2$ID
   == i & pharm.dat2$rownr > (entries-2)],
   extra))
   # the very last entry can be ignored since we add its pill days to the row before it
   # calculate adherence for all but the last 2 rows
   # assign a 1 if adherence is larger than 1 (in which case extra pill days were carried over
   # to the next row; this carrying over was done in the previous for-loop)
   # and otherwise use the adherence value as calculated
   pharm.dat2$adherence[pharm.dat2$ID == i & pharm.dat2$rownr < entries-1] <-
   ifelse(pharm.dat2$totdays[pharm.dat2$ID == i & pharm.dat2$rownr < entries-1] /
   pharm.dat2$intlength[pharm.dat2$ID == i & pharm.dat2$rownr < entries-1] > 1,
```

(*Continued*)

 © 2023. The Author(s).

Supplementary Table S2 (*Continued*)

```
    1,
    pharm.dat2$totdays[pharm.dat2$ID==i & pharm.dat2$rownr<entries-1] /
    pharm.dat2$intlength[pharm.dat2$ID==i & pharm.dat2$rownr<entries-1])
    # assign adherence to the final interval based on the adherence of the previous interval
    pharm.dat2$adherence[pharm.dat2$ID==i & pharm.dat2$rownr==(entries-1)] <-
    pharm.dat2$adherence[pharm.dat2$ID==i & pharm.dat2$rownr==(entries-2)]
    # calculate length of the final interval
    pharm.dat2$intlength[pharm.dat2$ID==i & pharm.dat2$rownr==(entries-1)] <-
    round(pharm.dat2$totdays[pharm.dat2$ID==i & pharm.dat2$rownr==(entries-1)] /
    pharm.dat2$adherence[pharm.dat2$ID==i & pharm.dat2$rownr==(entries-1)])
    # flag the last row for each patient for deletion
    pharm.dat2$remove[pharm.dat2$ID==i & pharm.dat2$rownr==entries] <- 1
}
# remove the last row for each patient since it does not contain useful information anymore
pharm.dat2 <- pharm.dat2[pharm.dat2$remove==0,]
pharm.dat2$remove <- NULL
# For each interval we have now calculated the adherence
# including the last interval
# but intervals do still overlap
## creating non-overlapping intervals ##
pharm.dat2$persontimeend <- pharm.dat2$persontimestart <- pharm.dat2$startdate <- pharm.dat2$enddate <-NA
for(i in unique(pharm.dat2$ID)) {
    entries <- length(pharm.dat2$ID[pharm.dat2$ID==i])
    pharm.dat2$startdate[pharm.dat2$ID==i & pharm.dat2$rownr==1] <-
    pharm.dat2$dispendate[pharm.dat2$ID==i & pharm.dat2$rownr==1]
    pharm.dat2$enddate[pharm.dat2$ID==i & pharm.dat2$rownr==1] <-
    as.character(as.Date(pharm.dat2$dispendate[pharm.dat2$ID==i & pharm.dat2$rownr==1]) +
    pharm.dat2$intlength[pharm.dat2$ID==i & pharm.dat2$rownr==1])
    for(rows in 2:entries) {
    pharm.dat2$startdate[pharm.dat2$ID==i & pharm.dat2$rownr==rows] <-
    as.character(as.Date(pharm.dat2$enddate[pharm.dat2$ID==i & pharm.dat2$rownr==(rows-1)])+1)
    daydiff <- as.Date(pharm.dat2$startdate[pharm.dat2$ID==i & pharm.dat2$rownr==rows]) -
    as.Date(pharm.dat2$dispendate[pharm.dat2$ID==i & pharm.dat2$rownr==rows])
    pharm.dat2$enddate[pharm.dat2$ID==i & pharm.dat2$rownr==rows] <-
    as.character(as.Date(pharm.dat2$startdate[pharm.dat2$ID==i & pharm.dat2$rownr==rows]) +
    (pharm.dat2$intlength[pharm.dat2$ID==i & pharm.dat2$rownr==rows] - daydiff))
    }
    pharm.dat2$persontimestart[pharm.dat2$ID==i] <-
    as.Date(pharm.dat2$startdate[pharm.dat2$ID==i]) -
    as.Date(pharm.dat2$startdate[pharm.dat2$ID==i & pharm.dat2$rownr==1])
    pharm.dat2$persontimeend[pharm.dat2$ID==i] <-
    as.Date(pharm.dat2$enddate[pharm.dat2$ID==i]) -
    as.Date(pharm.dat2$startdate[pharm.dat2$ID==i & pharm.dat2$rownr==1])
}
pharm.dat2$intlength <- pharm.dat2$totdays <- pharm.dat2$persontime <- pharm.dat2$dispendate <-
    pharm.dat2$pillsn <- pharm.dat2$dailydose <- NULL
# Now pharm.dat2 contains adherence per interval and no intervals overlap
# Adherence were manually calculated for the 180-days periods after index date
```

Note: DOAC adherence was evaluated by calculating PDC in persistent patients during a 180-day period after index date. We used an adapted version of the R Code published by Bijlsma et al to calculate PDC.[15]

 © 2023. The Author(s).

Supplementary Table S3 Factors associated with DOAC nonpersistence

Variable	Crude HR (95% CI)	Adjusted HR (95% CI)
TTR		
≥70%	Reference	Reference
<70%	1.07 (0.74–1.54)	1.09 (0.72–1.64)
TUR		
<4.2%	Reference	–
≥4.2% and <19.8%	0.91 (0.60–1.39)	
≥19.8%	1.15 (0.78–1.70)	
INR variability		
Low INR variability	Reference	Reference
Medium INR variability	1.13 (0.76–1.70)	1.13 (0.74–1.73)
High INR variability	0.95 (0.63–1.43)	0.97 (0.61–1.52)
Age		
<67 years	Reference	Reference
≥67 years and <80 years	0.74 (0.51–1.06)	0.76 (0.52–1.11)
≥80 years	1.10 (0.68–1.78)	1.20 (0.70–2.05)
Sex		
Female	Reference	Reference
Male	0.87 (0.62–1.21)	0.92 (0.65–1.32)
Frailty		
Frail patients	Reference	Reference
Nonfrail patients	1.05 (0.71–1.56)	0.99 (0.64–1.54)
VKA type		
Acenocoumarol	Reference	Reference
Phenprocoumon	3.02 (1.79–5.11)[a]	2.99 (1.74–5.12)[a]
Duration of previous VKA therapy (mo)	1.00 (0.99–1.01)	1.00 (1.00–1.01)
DOAC type		
Apixaban	Reference	Reference
Dabigatran	1.53 (1.03–2.28)[a]	1.68 (1.11–2.53)[a]
Edoxaban	NA	NA
Rivaroxaban	1.01 (0.65–1.55)	1.00 (0.65–1.55)
DOAC switch		
No switch	Reference	Reference
Switch	0.93 (0.53–162)	0.89 (0.50–1.60)
Concurrent APT use	0.96 (0.53–1.74)	0.91 (0.49–1.66)

Abbreviations: APT, antiplatelet therapy; CI, confidence interval; DOAC, direct oral anticoagulant; HR, hazard ratio; INR, international normalized ratio; NA, not applicable; TTR, time in therapeutic range; VKA, vitamin K therapy.
[a] p-Value < 0.05.

 © 2023. The Author(s).

Supplementary Table S4 INR control and DOAC nonpersistence, sensitivity analysis 1

Variable	VKA treatment <6 months ($n = 96$)	VKA treatment ≥ 6 months ($n = 341$)
	Crude HR (95% CI)	Crude HR (95% CI)
TTR		
≥70%	Reference	Reference
<70%	1.15 (0.53–2.48)	1.01 (0.66–1.55)
TUR		
<4.2%	Reference	Reference
≥4.2% and <19.8%	0.76 (0.32–1.77)	0.95 (0.58–1.55)
≥19.8%	1.12 (0.56–2.27)	1.12 (0.70–1.80)
INR variability		
Low INR variability	Reference	Reference
Medium INR variability	1.18 (0.50–2.79)	1.05 (0.65–1.67)
High INR variability	0.75 (0.32–1.74)	0.95 (0.59–1.55)

Abbreviations: CI, confidence interval; HR, hazard ratio; INR, international normalized ratio; TTR, time in therapeutic range; TUR, time under therapeutic range.
Note: Evaluation of INR control and DOAC nonpersistence, stratified by VKA duration.

Supplementary Table S5 INR control and DOAC nonpersistence, sensitivity analysis 2

Variable	Crude HR (95% CI)
TTR	
≥70%	Reference
<70%	1.08 (0.7–1.58)
TUR	
<4.2%	Reference
≥4.2% and <19.8%	0.91 (0.59–1.39)
≥19.8%	1.15 (0.78–1.70)
INR variability	
Low INR variability	Reference
Medium INR variability	1.14 (0.76–1.71)
High INR variability	0.96 (0.64–1.44)

Abbreviations: CI, confidence interval; HR, hazard ratio; INR, international normalized ratio; TTR, time in therapeutic range; TUR, time under therapeutic range.
Note: Evaluation of INR control and DOAC nonpersistence with a more strict definition of nonpersistence (i.e., a gap of more than 90 days after the end of the last DOAC prescription). $N = 437$.

 © 2023. The Author(s).

Supplementary Table S6 INR control and DOAC nonpersistence, sensitivity analysis 3

Variable	Crude HR (95% CI)
TTR	
≥70%	Reference
<70%	1.02 (0.66–1.45)
TUR	
<4.2%	Reference
≥4.2% and <19.8%	1.04 (0.62–1.50)
≥19.8%	0.99 (0.66–1.55)
INR variability	
Low INR variability	Reference
Medium INR variability	1.16 (0.74–1.80)
High INR variability	0.98 (0.63–1.52)

Abbreviations: CI, confidence interval; HR, hazard ratio; INR, international normalized ratio; TTR, time in therapeutic range; TUR, time under therapeutic range.
Note: Evaluation of INR control and DOAC nonpersistence in patients with atrial fibrillation ($n = 356$).

Supplementary Table S7 INR control and DOAC nonadherence, sensitivity analysis 1

Variable	VKA treatment < 6 months ($n = 96$)	VKA treatment ≥ 6 months ($n = 341$)
	Crude OR (95% CI)	Crude OR (95% CI)
TTR		
≥70%	Reference	Reference
<70%	1.84 (0.21–16.02)	1.36 (0.63–2.95)
TUR		
<4.2%	Reference	Reference
≥4.2% and <19.8%	0.33 (0.03–3.47)	0.51 (0.21–1.26)
≥19.8%	0.80 (0.16–3.93)	0.91 (0.40–2.05)
INR variability		
Low INR variability	Reference	Reference
Medium INR variability	0.29 (0.04–2.01)	0.68 (0.29–1.63)
High INR variability	0.23 (0.04–1.33)	0.95 (0.42–2.19)

Abbreviations: CI, confidence interval; INR, international normalized ratio; OR, odds ratio; TTR, time in therapeutic range; TUR, time under therapeutic range.
Note: Evaluation of INR control and DOAC nonadherence, stratified by VKA duration.

Supplementary Table S8 INR control and DOAC nonadherence, sensitivity analysis 2

Variable	Crude OR (95% CI)
TTR	
≥70%	Reference
<70%	1.51 (0.63–3.65)
TUR	
<4.2%	Reference
≥4.2% and <19.8%	0.49 (0.19–1.24)
≥19.8%	0.56 (0.23–1.37)
INR variability	
Low INR variability	Reference
Medium INR variability	0.49 (0.18–1.35)
High INR variability	0.80 (0.34–1.86)

Abbreviations: CI, confidence interval; INR, international normalized ratio; NA, not applicable; OR, odds ratio; TTR, time in therapeutic range; TUR, time under therapeutic range.
Note: Evaluation of INR control and DOAC nonadherence for time period of 0–6 months, in patients with atrial fibrillation ($n = 356$).

 © 2023. The Author(s).

Supplementary Table S9 Differences between adherent and nonadherent patients

Patient characteristic	Adherent patients ($n = 363$)	Nonadherent patients ($n = 43$)	p-Value
Age (years), mean ± SD	69.6 ± 12.0	69.8 ± 15.5	0.94
Male, no. (%)	196 (54.0)	26 (60.5)	0.52
Indication anticoagulation therapy, no. (%)			
AF	298 (82.5)	30 (69.8)	0.14
VTE	42 (11.6)	12 (27.9)	
VKA treatment, no. (%)			
Acenocoumarol	345 (95.0)	40 (93.0)	0.48
Phenprocoumon	15 (5.0)	3 (7.0)	
VKA target range, no. (%)			
2.0–3.0	–	–	0.70
2.5–3.5			
Duration of previous VKA therapy (months), median [IQR]	33.2 [7.6–62.3]	25.9 [7.2–57.6]	0.89
Previous periods of VKA treatment, no. (%)	26 (7.2)	6 (14.0)	0.13
Frailty[a], no. (%)			
Frail patients	93 (25.6)	9 (20.9)	0.63
Nonfrail patients	270 (74.4)	34 (79.1)	
DOAC treatment at index date, no. (%)			
Apixaban	124 (34.2)	15 (34.9)	0.85
Dabigatran	98 (27.0)	14 (32.6)	
Edoxaban	–	–	
Rivaroxaban	123 (33.9)	13 (30.2)	
DOAC switch after index date, no. (%)			
Switch	32 (8.8)	5 (11.6)	0.57
No switch	331 (91.2)	38 (88.4)	
APT use at index date, no. (%)	34 (9.4)	3 (7.0)	0.78
TTR, median [IQR]	57.8 [41.7–73.1]	50.0 [32.5–70.7]	0.24
<70%, no. (%)	246 (67.8)	32 (74.4)	
≥70%, no (%)	117 (32.2)	11 (25.6)	
TUR, median [IQR]	12.2 [1.5–25.0]	10.0 [0.0–33.5]	0.82
INR variability, median [IQR]	0.26 [0.16–0.43]	0.22 [0.15–0.40]	0.40

Abbreviations: AF, atrial fibrillation; APT, antiplatelet therapy, DOAC, direct oral anticoagulant; INR, international normalized ratio; TTR, time in therapeutic range; TUR, time under therapeutic range; VKA, vitamin K Therapy; VTE, venous thromboembolism.
[a]Patients with INR values measured by a health care professional at home were defined as frail, in contrast to patients with INR measurements at the outpatient clinic or INR measurements by the patients themselves (i.e., self-testing).

 © 2023. The Author(s).

Supplementary Table S10 INR control and DOAC nonadherence

	Crude OR				Adjusted OR			
Variable	0–6 months (n = 406)	0–1 year (n = 302)	0–2 years (n = 217)	0–3 years (n = 150)	0–6 months (n = 406)	0–1 year (n = 302)	0–2 years (n = 217)	0–3 years (n = 150)
TTR								
≥70%	Reference	Reference	Reference	NA	Reference	Reference	Reference	NA
<70%	1.38 (0.67–2.84)	1.29 (0.55–3.01)	1.86 (0.60–5.73)		1.59 (0.72–3.52)	1.90 (0.73–4.98)	2.28 (0.66–7.88)	
TUR								
<4.2%	Reference	Reference	Reference	Reference	–	–	–	–
≥4.2% and <19.8%	0.48 (0.21–1.12)	0.62 (0.23–1.65)	0.94 (0.30–2.94)	4.00 (0.40–39.82)				
≥19.8%	0.87 (0.43–1.79)	1.09 (0.47–2.56)	1.48 (0.52–4.20)	9.56 (1.13–80.73)[a]				
INR variability								
Low INR variability	Reference	Reference	Reference	Reference	Reference	Reference	Reference	Reference
Medium INR variability	0.61 (0.28–1.35)	0.92 (0.38–2.19)	1.00 (0.33–3.02)	1.21 (0.23–6.31)	0.55 (0.24–1.28)	0.80 (0.31–2.09)	0.90 (0.27–3.02)	0.99 (0.14–7.04)
High INR variability	0.73 (0.35–1.53)	0.59 (0.23–1.50)	0.97 (0.33–2.82)	1.51 (0.34–6.65)	0.64 (0.28–1.47)	0.49 (0.17–1.42)	0.81 (0.25–2.69)	1.02 (0.17–6.02)
Age								
<67 years	Reference	Reference	Reference	Reference	Reference	Reference	Reference	Reference
≥67 years and <80 years	0.82 (0.39–1.69)	0.37 (0.16–0.86)[a]	0.67 (0.26–1.73)	0.28 (0.07–1.16)	0.89 (0.42–1.90)	0.41 (0.17–1.01)	0.83 (0.30–2.32)	0.41 (0.07–2.32)
≥80 years	1.30 (0.55–3.10)	0.87 (0.31–2.43)	1.16 (0.28–4.71)	1.15 (0.21–6.41)	1.70 (0.62–4.65)	1.22 (0.37–3.96)	1.70 (0.34–8.59)	1.62 (0.16–16.37)
Sex								
Female	Reference	Reference	Reference	Reference	Reference	Reference	Reference	Reference
Male	1.30 (0.68–2.48)	1.75 (0.79–3.85)	2.10 (0.79–5.61)	9.58 (1.19–76.84)	1.37 (0.69–2.71)	1.38 (0.58–3.28)	2.00 (0.70–5.71)	11.78 (1.28–10.86)
Frailty								
Frail patients	Reference	Reference	Reference	Reference	Reference	Reference	Reference	Reference
Nonfrail patients	0.77 (0.36–1.66)	0.61 (0.22–1.65)	0.83 (0.27–2.60)	0.77 (0.16–3.77)	0.62 (0.25–1.52)	0.62 (0.20–1.92)	0.77 (0.21–2.81)	1.26 (0.16–10.04)
VKA type								
Acenocoumarol	Reference	Reference	NA	NA	Reference	Reference	NA	NA
Phenprocoumon	1.44 (0.41–5.09)	1.49 (0.32–6.98)			1.59 (0.44–5.82)	1.91 (0.37–9.96)		
Duration of previous VKA therapy (in months)	1.00 (0.99–1.01)	1.00 (0.99–1.01)	0.99 (0.98–1.01)	0.99 (0.92 1.02)	1.00 (0.99–1.01)	1.00 (0.99–1.01)	0.99 (0.98–1.01)	0.99 (0.97–1.01)
DOAC type								
Apixaban	Reference	Reference	Reference	Reference	Reference	Reference	Reference	Reference
Edoxaban	1.18 (0.54–2.56)	2.68 (1.06–6.75)	1.29 (0.46–3.57)	1.29 (0.46–3.57)	1.12 (0.50–2.50)	2.98 (1.12–7.90)[a]	1.77 (0.59–5.31)	1.30 (0.21–8.12)

 © 2023. The Author(s).

Supplementary Table S10 (*Continued*)

Dabigatran	0.46 (0.06–3.69)	1.63 (0.32–8.44)	NA	NA	0.42 (0.05–3.47)	1.97 (0.35–10.93)	NA	NA
Rivaroxaban	0.87 (0.40–1.91)	0.91 (0.32–2.61)	0.65 (0.21–2.03)	0.65 (0.21–2.03)	0.87 (0.39–1.95)	0.87 (0.30–2.57)	0.72 (0.22–2.37)	1.23 (0.22–7.05)
DOAC switch								
No switch	Reference	Reference	NA	NA	Reference	Reference	NA	NA
Switch	1.36 (0.50–3.70)	0.65 (0.15–2.88)			1.40 (0.49–3.99)	0.66 (0.14–3.18)		
Concurrent APT use	0.73 (0.21–2.47)	2.07 (0.73–5.91)	1.20 (0.26–5.63)	1.16 (0.14–9.95)	0.72 (0.20–2.56)	1.52 (0.49–4.77)	1.07 (0.21–5.55)	2.77 (0.18–42.74)

Abbreviations: APT, antiplatelet therapy; CI, confidence interval; DOAC, direct oral anticoagulant; INR, international normalized ratio; NA, not applicable; PDC, proportion of days covered; TTR, time in therapeutic range; VKA, vitamin K antagonist.
Note: Logistic regression analyses evaluating the association between INR control and DOAC nonadherence. Nonadherence was defined as a PDC value below 90%. Because of collinearity, TUR was not included in the adjusted models.
[a]p-Value < 0.05.

 © 2023. The Author(s).
